# Cardiotoxic Effects of *Lachesis acrochorda* Snake Venom in Anesthetized Wistar Rats

**DOI:** 10.3390/toxins16090377

**Published:** 2024-08-28

**Authors:** Karen Leonor Ángel-Camilo, Mary Luz Bueno-Ospina, Ivonne Carolina Bolaños Burgos, Santiago Ayerbe-González, José Beltrán-Vidal, Ana Acosta, Jaime Álvarez-Soler, Jimmy Alexander Guerrero-Vargas

**Affiliations:** 1 Grupo de Investigaciones Herpetológicas y Toxinológicas (GIHT), Centro de Investigaciones Biomédicas—Bioterio (CIBUC-Bioterio), Departamento de Biología, Facultad de Ciencias Naturales, Exactas y de la Educación, Universidad del Cauca, Popayán 190003, Colombia; klangel@unicauca.edu.co (K.L.Á.-C.); carobol12@gmail.com (I.C.B.B.); sayerbe@hotmail.com (S.A.-G.); jbeltran@unicauca.edu.co (J.B.-V.); 2 Programas de Pos-Graduação em Ciências Farmacêuticas, Faculdade de Farmácias, Odontologia e Enfermagem, Universidade Federal do Ceará, Fortaleza 60020-181, Brazil; 3 Grupo de Investigaciones Herpetológicas y Toxinológicas (GIHT), Centro de Investigaciones Biomédicas—Bioterio (CIBUC-Bioterio), Programa de Maestría en Biología, Facultad de Ciencias Naturales, Exactas y de la Educación, Universidad del Cauca, Popayán 190003, Colombia; mlbueno@unicauca.edu.co; 4 Faculdade de Medicina, Universidade Federal de Minas Gerais, Belo Horizonte 31270-901, Brazil; 5 Grupo de Investigación en Producción y Sanidad en Ciencias Veterinarias y Zootecnias (PROSAVEZ), Facultad de Medicina Veterinaria y Zootecnia, Fundación Universitaria San Martín, Cali 760001, Colombia; ana.acosta@sanmartin.edu.co; 6 Grupo de Investigación en Inmunología y Enfermedades Infecciosas, Departamento de Patología, Facultad de Ciencias de la Salud, Universidad del Cauca, Popayán 190003, Colombia; jalvarezs@unicauca.edu.co; 7 Grupo de Investigaciones Herpetológicas y Toxinológicas (GIHT), Centro de Investigaciones Biomédicas—Bioterio (CIBUC-Bioterio), Museo de Historia Natural, Departamento de Biología, Doctorado en Ciencias Ambientales, Facultad de Ciencias Naturales, Exactas y de la Educación, Universidad del Cauca, Popayán 190003, Colombia

**Keywords:** cardiotoxic, *Lachesis acrochorda*, snake venom, *Rattus norvegicus*

## Abstract

Ophidism is a public health problem in tropical countries, occurring predominantly in rural areas. In Colombia, among the species responsible for snakebite envenomation, inflicting high mortality, is the Chocoan bushmaster, *Lachesis acrochorda*, better known locally by the names “*verrugosa* (warty)” and “*pudridora* (rot-causing)”. In this research, the cardiotoxic effect of the venom of *L. acrochorda* in male Wistar rats weighing 230 ± 20 g was evaluated. A statistical design of randomized blocks was implemented with three treated groups, injected with lyophilized venom (doses of 3.22 μg/g, 6.43 μg/g, 12.86 μg/g), and a control group injected with 0.9% saline solution. Electrocardiographic (ECG) recordings were taken from the anesthetized animals, revealing an increase in the amplitude of the P and T waves and an increase in the duration of the QT intervals in the electrocardiographic recordings. These increases were not observed in the control biomodels. In the analysis of the CK and CK-MB enzyme levels, increases were also observed in the levels of cardiac isoenzymes in the injected animals, but none in the control animals. The histopathological analyses carried out reveal that the injected animals showed effects such as interfibrillar and perivascular edema, cellular shortening of the cardiomyocytes, foci with tissue destructuring, and necrosis with contraction bands. In conclusion, the venom of the *Lachesis acrochorda* snake increases the P and T waves and the QT interval and increases the CK and CK-MB enzymes in the blood. Additionally, it causes interfibrillar and perivascular edema in the cardiac tissue, cardiocytolysis, and contraction bands.

## 1. Introduction

Due to its geographical location and climatic characteristics [1], Colombia has a wide variety of venomous snakes that cause snakebite envenomation (SBE). Countries in Sub-Saharan Africa, South Asia, and other parts of Latin America are among other territories mainly affected by this public health problem [1,2].

Snakes of the genus *Lachesis* are the largest vipers in the world and can inject large doses of venom with their long fangs when they bite [3]. Their venom has been reported to produce local manifestations such as intense pain, paresthesias, hypothermia, progressive edema, bleeding (ecchymosis, flictenas), coliquative subepidermal and muscular necrosis, regional lymphadenitis and lymphangitis, compartment syndrome, and rapid onset myonecrosis. It can further produce systemic manifestations such as severe coagulopathy with fibrinogen depletion (hemorrhage); intravascular coagulation with thrombosis (retinal arteries, renal vessels, hemorrhoidal veins) due to erythrocyte agglutination; thrombin-like procoagulant action, bradycardia, arterial hypotension, abdominal pain, diarrhea, nausea, vomiting, sweating, and necrosis. These manifestations can result in permanent after-effects or even death [4,5,6,7,8,9,10,11].

*L. acrochorda* is distributed along the Atlantic and Pacific coasts and on the western slopes of Panama, along the Atlantic coast in northwest Colombia, extending southward through the lowlands of the Cauca and Magdalena River valleys, and along the Pacific slope of Colombia into northwest Ecuador [8,12,13].

As a public health problem, SBE has traditionally been inadequately measured, since epidemiological surveillance, the production of antivenoms, and knowledge regarding prevention and treatment in the event of cases are limited. This type of accident thus becomes a risk for society. *L. acrochorda* produces between 2–3% of the recorded bites, with a high mortality [14]. The objective of this research was to determine the cardiotoxic effect of *L. acrochorda* venom by means of electrocardiographic recordings and enzymatic and histopathological analyses.

## 2. Results

The results demonstrate cardiac effects produced in the biomodels injected intraperitoneally with the venom of the *Lachesis acrochorda* snake (SVLa). These include an increase in the amplitude of the P and T waves and an increase in the duration of the QT intervals in the electrocardiographic recordings, which were not observed in the control biomodels. Increases in the levels of cardiac isoenzymes were also observed in injected animals but not in the control animals. The histopathological analyses carried out reveal that the injected animals showed effects such as interfibrillar and perivascular edema, cellular shortening of the cardiomyocytes, foci with tissue destructuring, and necrosis with contraction bands.

### 2.1. Electrocardiographic Analysis

**Figure 1:**
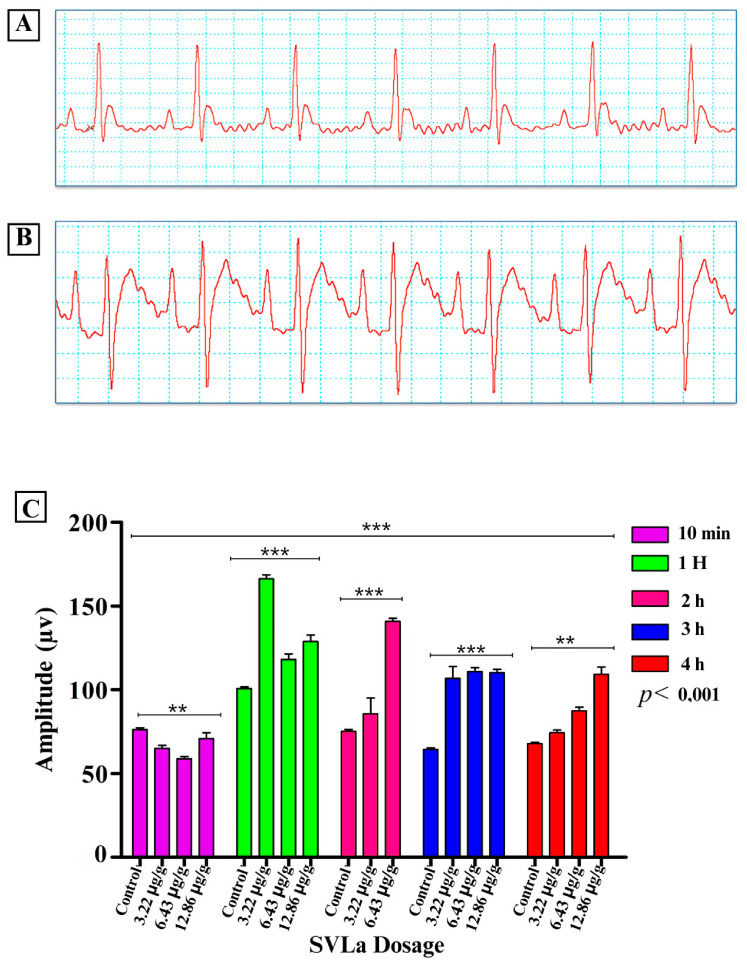
Alterations in the P wave. (**A**) Normal ECG recording in a control rat; (**B**) ECG recording in rats injected with *L. acrochorda* venom (SVLa), indicating the abnormal elevation of the P wave; (**C**) two-way ANOVA statistical analysis, comparing the values of the P wave amplitude across the time blocks (hours) and doses of SVLa with those of the control group. ** Highly significant difference, *** Extremely significant difference.

**Figure 2:**
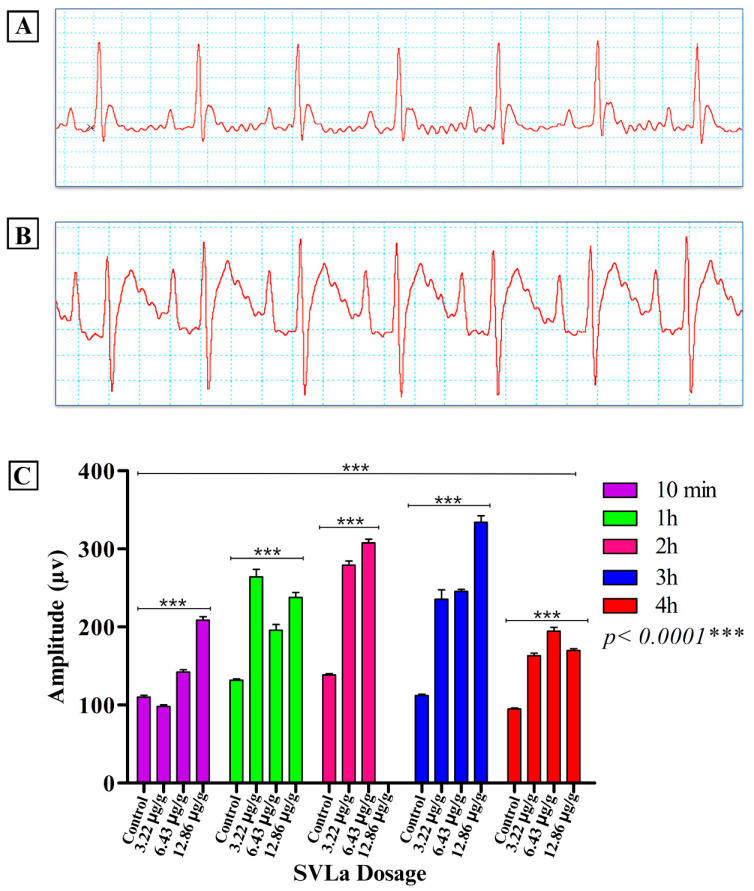
Alterations in the T wave. (**A**) Normal ECG recording in a control rat; (**B**) ECG recording in rats injected with SVLa, indicating increased T wave; (**C**) two-way ANOVA statistical analysis. *** Extremely significant difference.

**Figure 3:**
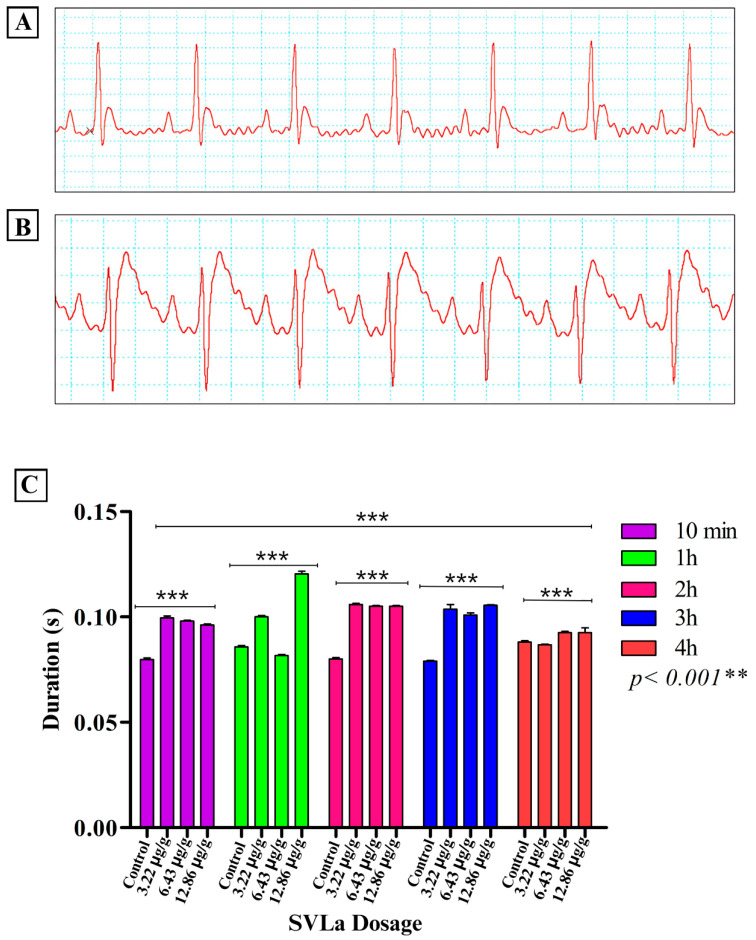
Alterations in the duration of the QT interval. (**A**) Normal ECG recording in a control rat; (**B**) ECG recording in rats injected with SVLa, indicating increased QT interval; (**C**) two-way ANOVA statistical analysis, contrasting the values of the T wave amplitude in each of the time blocks (hours) and groups (control and SVLa doses of 3.22 μg/g, 6.43 μg/g, 12.86 μg/g), showing a highly significant difference (*p* < 0.005) between the T wave amplitude of the controls and that of the dosed ones, with the greatest elevation occurring at 2 and 3 h. ** Highly significant difference, *** Extremely significant difference.

**Figure 4:**
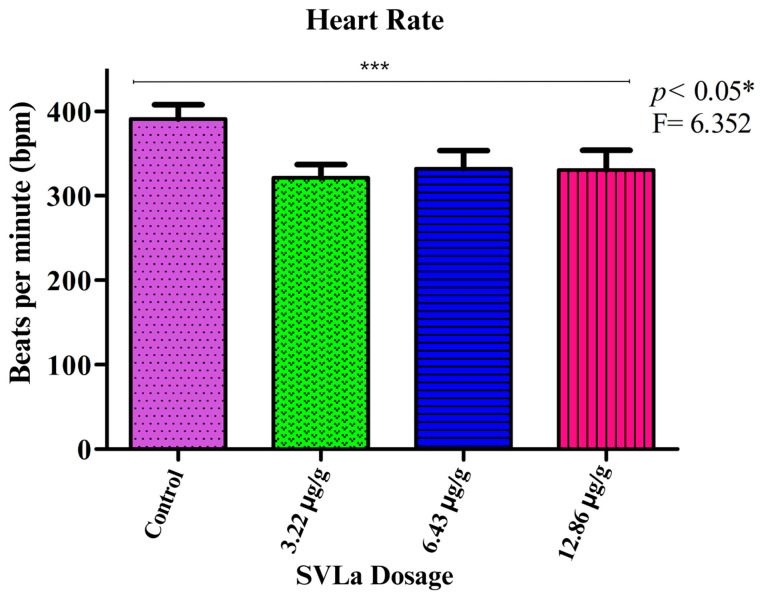
Alterations in heart rate. A one-way ANOVA analysis was conducted for the heart rate shown by the rats in the control group and those receiving doses of SVLa (3.22 μg/g, 6.43 μg/g, 12.86 μg/g), after performing Dunnett’s test. * Significant difference, *** Extremely significant difference. Contrl group purple bar, dose of 0.22 μg/g green bar, dose of 6.43 μg/g blue bar, dose of 12.86 μg/g red bar.

Cardiac alterations were reported in the electrocardiographic recordings, clearly observed in the electrocardiographic variations in the control group compared with those receiving doses of SVLa (3.22 μg/g, 6.43 μg/g, 12.86 μg/g). These alterations were notable in the recordings of the amplitude of the P and T waves (Figure 1 and Figure 2, respectively), which, together with the Q-T segment (Figure 3), showed significant increases compared with the recording from the control group. After the application of each of the doses used, as seen in Figure 3, the heart rate decreased in the groups receiving the doses in relation to the control group (Figure 4).

### 2.2. Analysis of Cardiac Enzymes CK and CK-MB

**Figure 5:**
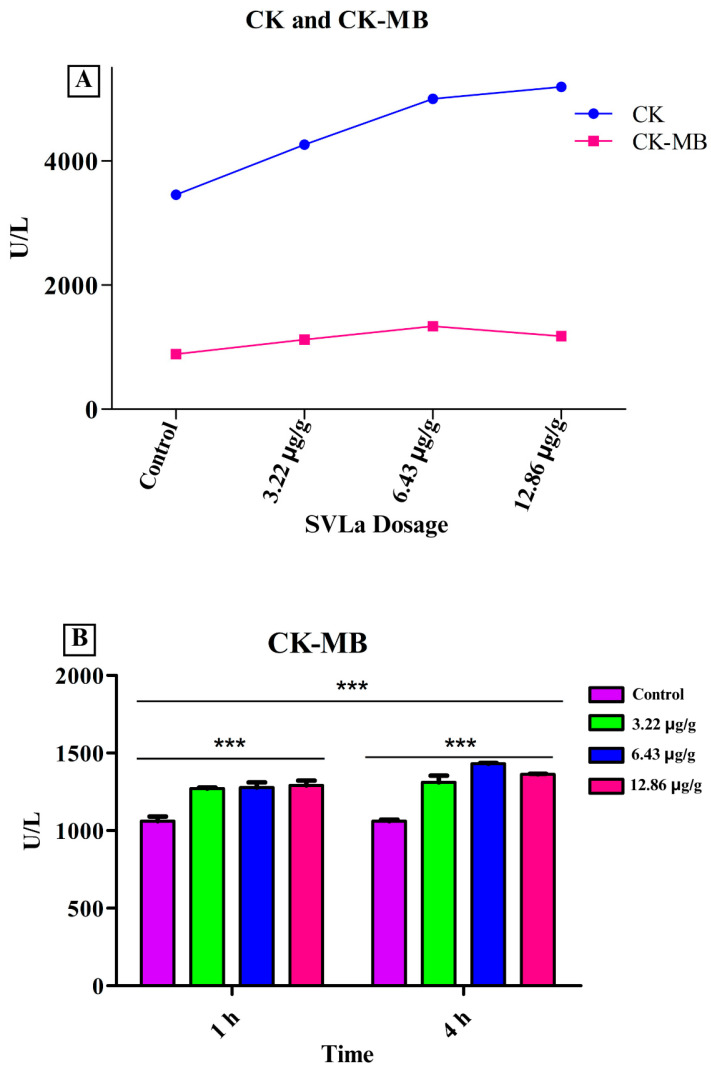
Striated muscle (CK) and myocardial fraction (CK-MB) enzymes. (**A**) CK and CK-MB values for each of the doses used; (**B**) two-way ANOVA contrast of the blood concentration values of the CK-MB isoenzyme at the first and fourth hours after inoculation with SVLa. *** Extremely significant difference.

In order to strengthen the evidence of the myotoxic and cardiotoxic effects caused by SVLa, levels of the CK and CK-MB enzymes were measured in the blood of the experimental biomodels. These results are shown in Figure 5, in which a trend toward increasing CK and CK-MB enzyme levels can be observed, suggesting myocardial damage.

### 2.3. Histopathological Analysis

**Figure 6:**
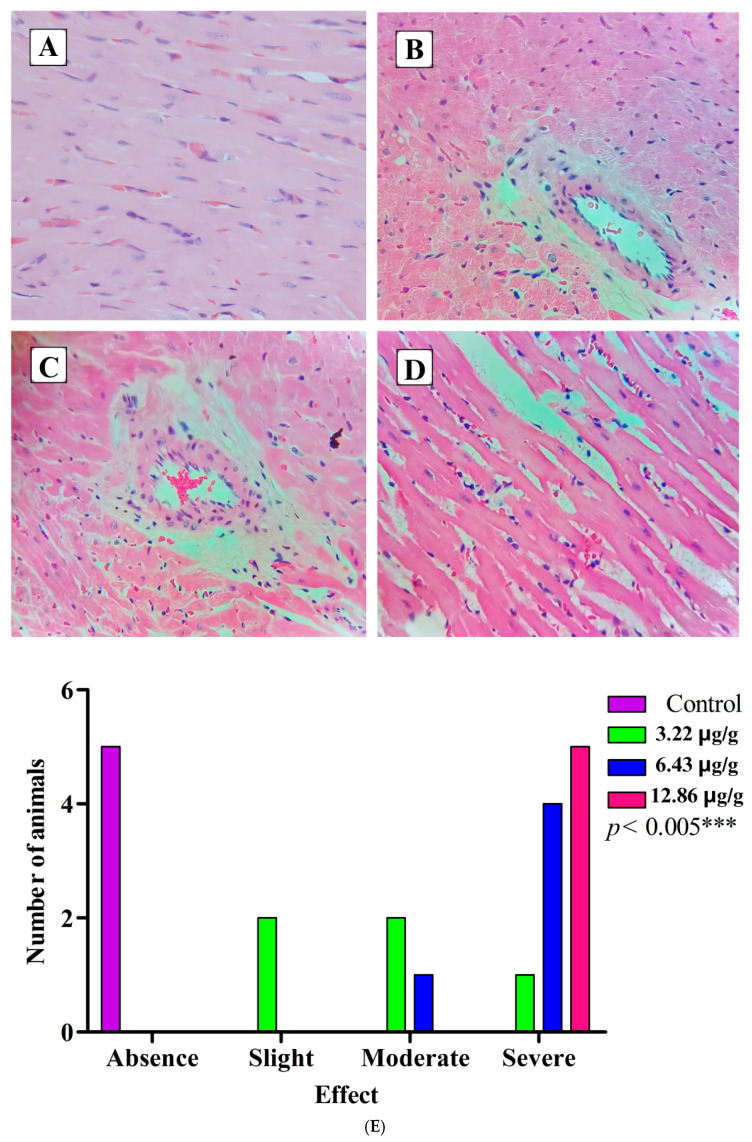
Interfibrillar edema. (**A**) Normal tissue from control rats; (**B**) tissue with the presence of mild interfibrillar edema (rats injected with a dose of SVLa at 3.22 μg/g); (**C**) tissue with the presence of moderate interfibrillar edema (rats injected with a dose of SVLa at 6.43 μg/g); (**D**) tissue with severe interfibrillar edema (rats injected with a dose of SVLa at 12.86 μg/g), H-E 40X; (**E**) statistical analysis of edema by the Chi-squared test. *** Extremely significant difference.

**Figure 7:**
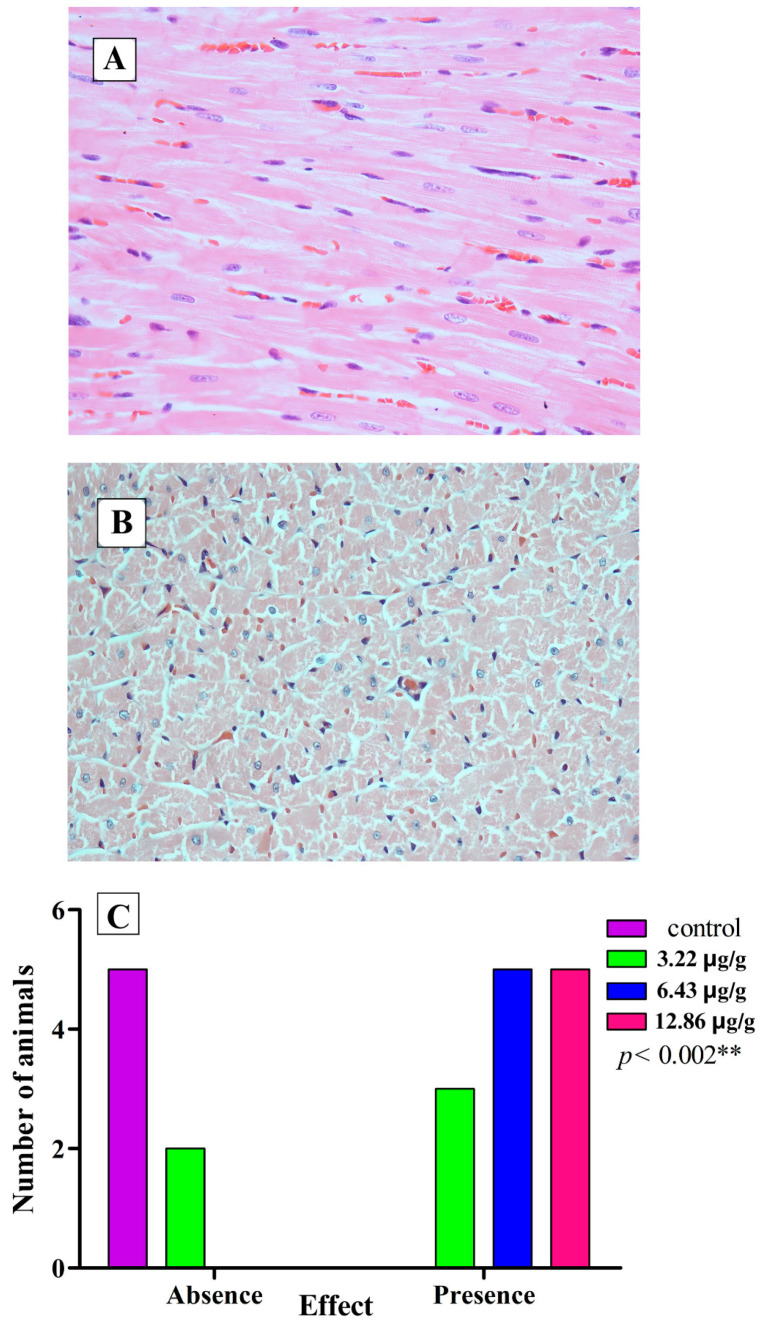
Cardiocytolysis. (**A**) Normal tissue from control rats; (**B**) tissue with destructuring, necrosis with contraction bands, H-E 40X; (**C**) statistical analysis of the presence of cardiocytolysis by the Chi-squared test. ** Highly significant difference.

**Figure 8:**
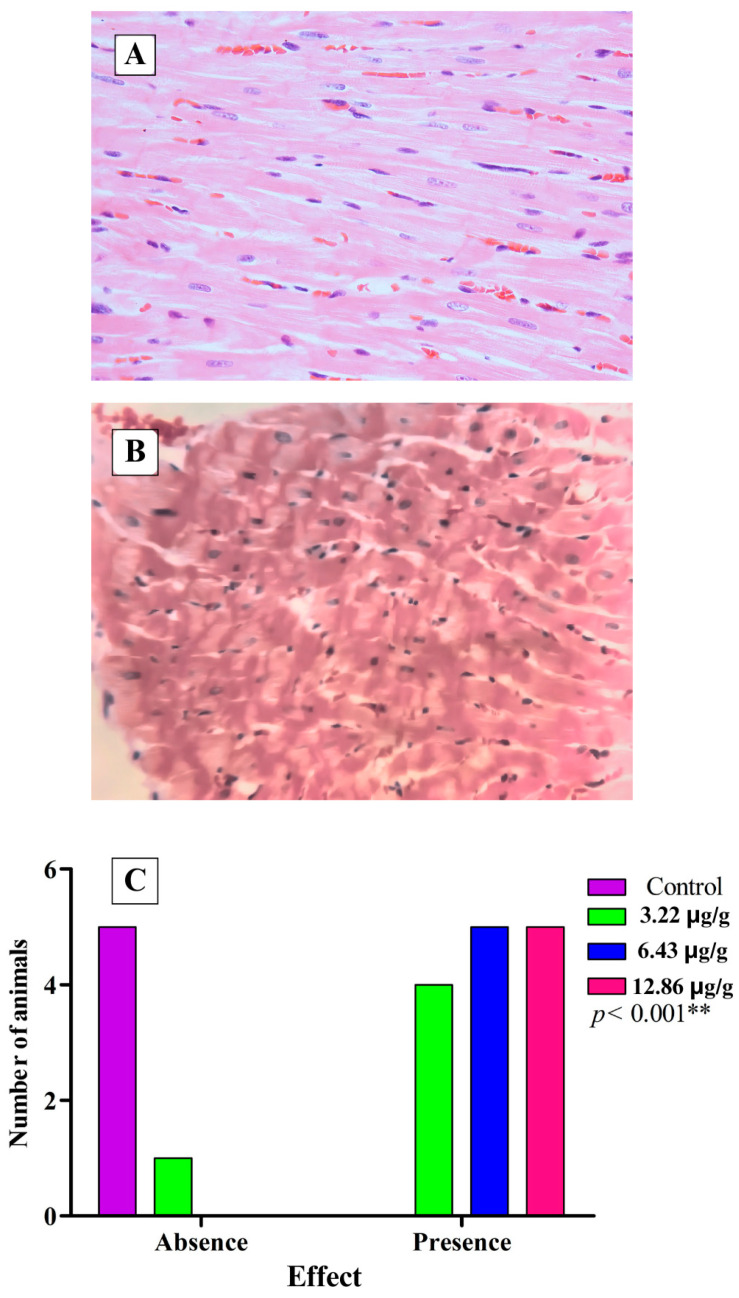
Contraction bands. (**A**) normal tissue from control rats; (**B**) tissue with disorganization of its cytoarchitecture, shortened cardiomyocytes, and contraction bands, H-E 40X; (**C**) statistical analysis of the presence of contraction bands by the Chi-squared test, indicating that the level of alteration depends on the dose applied. ** Highly significant difference.

In addition to the electrocardiographic recordings and the measurement of the levels of CK and CK-MB in the blood of the experimental biomodels, the heart was extracted from the biomodels, and sections of the cardiac tissues were prepared, revealing such alterations as interfibrillar edema (Figure 6), cardiocytolysis (Figure 7), and contraction bands (Figure 8) to confirm the cardiotoxic effect caused by the venom of *L. acrochorda*. Additionally, in Figure 6B,C, the presence of mild to moderate perivascular edema may also be seen.

## 3. Discussion

Envenomation caused by species of the genus *Lachesis* features both local and systemic pathophysiological effects. Local effects include cytotoxic (blistering edema, flictena), hemotoxic (ecchymosis), and myotoxic effects (necrosis of striated muscles), while systemic effects include the anticoagulant (hemorrhages) and coagulant (arterial and venous thrombosis) effects [7,8,9,10,11], as well as effects such as the stimulation of platelet aggregation processes [3]. This venom also produces, indirectly, kallicrein and prekallikrein (Fletcher factor), which causes increased peristalsis, and directly produces bradykinins, responsible for hypotension, diarrhea, and bradycardia [15,16,17,18]. The aforementioned may account for the heart failures demonstrated in this work in the biomodels injected with SVLa (Figure 1, Figure 2 and Figure 3), which are likewise symptoms present in patients who are victims of envenomation due to bites by snakes of the genus *Lachesis*. This increases the probability of death for the patients.

The electrocardiographic analysis used, as references, the amplitude of the P and T waves, the duration of the QT interval, and the heart rate. In relation to the P wave, which represents atrial activation [19], a statistically significant elevation (*p* < 0.001) was observed when comparing the amplitudes of this wave for the control biomodels and those injected with venom. This shows a cardiac alteration (Figure 5) that is related to an increase in the size of the left atrium of the heart, possibly due to the work overload of the heart when pumping clotted blood. Regarding the T wave, which represents ventricular repolarization [19], the amplitude of this wave in Wistar rats typically ranges from 130 to 230 µv [20]. A statistically significant elevation (*p* < 0.001) of the T wave was observed in the biomodels injected with the established doses of venom (Figure 2). One of the possible causes of elevated T wave amplitude may be an electrolyte disorder known as hyperkalemia, which involves elevated potassium [K+] levels in the blood [21]. This is based on the fact that some authors claim that the effects on the ECG caused by hyperkalemia generate the elevation of the T segment, masking or simulating an infarction [22,23,24,25,26,27]. Potassium is one of the most important electrolytes in specific and non-specific myocardial tissue [24], which is explained by the fact that potassium is important in the repolarization of the action potential of cardiac cells. When blood potassium increases, there is greater cellular excitability since the extracellular potassium lowers the potential difference between the interior and exterior of the cardiac cells, altering repolarization and causing the cells to be more excitable, which is expressed in the electrocardiographic record with the presence of a tall and peaked T wave [26]. In fact, the increase in potassium leads to a decrease in the action potential, with a gradual increase towards less negative values. These events can cause tall, sharp T waves, reduced P wave amplitude, prolongation of the PQ interval, enlargement of the QRS complex, and ST segment elevation [22]. Additionally, the elevation of the ST segment may be related to ischemia prior to the development of a myocardial infarction, which was confirmed by enzymatic and histological analyses.

Moreover, the duration of the QT interval, which represents cardiac post-depolarization, was analyzed. A statistically significant increase (*p* < 0.05) in the duration of the QT interval was observed when comparing the recordings of the control biomodels with those that received subdoses of venom. This alteration may be due to the blockage of potassium channels in the action potential of the cells, which leads to the prolongation of the repolarization phase. This, acting as an adverse effect, is manifested by QT prolongation in the electrocardiogram [28], thereby producing bradycardia (Figure 3). This is consistent with the results shown in Figure 4.

The procoagulant activity of this venom was evident in this work, although other studies suggest that the venom of snakes of the genus *Lachesis* produces severe coagulopathies and hemorrhages [4,7]. In this study, it was not observed that the venom of *L. acrochorda* had this characteristic, since biomodels injected with higher doses of venom showed more blood coagulation, thus suggesting that the venom of this snake species is not hemorrhagic, which aligns with what was reported previously [3]. This is largely due to the levels of serine proteases present in this venom [3,12], which favor the processes of platelet aggregation and clot formation. These results differ greatly from the studies carried out with species such as *Bothrops jararaca*, where the platelet aggregation processes reported for this species are much less than those reported for SVLa [29,30].

Another parameter analyzed was the levels of the CK-MB isoenzyme. The results showed statistically significant differences between the CK-MB values of the control group and the groups receiving doses of venom (*p* > 0.05), with the first and fourth hours being the times in which the greatest elevation of the CK-MB isoenzyme was observed, caused by the venom of *L. acrochorda* (Figure 5). This indicates alterations in cardiac function such as myocardial infarction [31] or cardiovascular alterations, as also reported for the venom of *Lachesis muta* in Brazil [32].

The elevation of the CK was greater than that of the MB fraction, indicating the strong myotoxic activity of the metalloproteinases on the striated muscles (rhabdomyolysis) at the injection site and systemically. This activity causes the release of myoglobin into the bloodstream and affects kidney function by producing failure in the glomerular filtration, due to myoglobinuria with acute renal failure. Furthermore, due to the cytotoxic activity of the venom, there is direct damage by the venom to the endothelium in the small vessels of the kidney, with deposits of fibrin [33].

In the histopathological study, a number of alterations were found, which led to the confirmation of the alterations encountered in the ECG and in the levels of CK and CK-MB. Regarding the hemorrhage present in some tissues, although this is not statistically significant, it may be due to an effect of snake venom metalloproteinases (SVMPs), which physiologically have hemorrhagic effects [34] and are present in the SVLa [12]. Other reports have also mentioned that the venom of *L. acrochorda* has a hemorrhagic and coagulant effect [35].

The biomodels subjected to injected with *L. acrochorda* venom suffered from edema. A clear example was at the 12.86 μg/g dose, in which the edema was found in greater quantity and diameter compared to those in the tissue of the rats inoculated with the 6.43 μg/g dose of the venom, which showed this alteration in some regions of the tissue more than in others. At the 3.22 μg/g dose, few edema foci were found, although these tended to increase over time (Figure 6). Perivascular edema was also among the effects, suggesting that the venom affects the vascular composition of the tissues and the circulation of the blood. The mechanism that could explain the formation of edema in the cardiac tissue is related to the initial process of inflammation, vasodilation of the arterioles, and the increase in blood flow, which together increase the intravascular hydrostatic pressure, leading to the passage of fluid from the capillaries to the tissues [36]. This may be related to a possible electrolyte disorder produced by the venom of *L. acrochorda*.

Tissue lesions were also found, such as cardiocytolysis (Figure 8) and necrosis with contraction bands (Figure 8), which showed statistically significant differences (*p* < 0.001) between the control group and the groups injected with SVLa. This suggests that *L. acrochorda* envenomation affects the cardiac system, directly or indirectly.

Another possible cause of the effects produced by this venom may be that because it was collected from specimens of *L. acrochorda* that live only in the department of Cauca, the venom used in this study has a different composition. This would lead us to consider that the situation is similar to that of the *Bothrops asper* species, in which the composition of the venom varies according to the geographical distribution of the organisms [37,38,39].

The results of this work provide a tool for physicians and decision-making institutions in the health sector to incorporate into clinical treatment protocols for *Lachesis* snakebite, particularly that caused by *Lachesis acrochorda*, the performance of electrocardiograms, analysis of CK, CK-MB, and troponin enzymes, and constant monitoring of cardiac activity. From the point of view of biotechnological or pharmacological application, these results suggest researching the components of *L. acrochorda* venom that cause decreased heart rate as possible antiarrhythmics in paroxysmal tachycardia, atrial fibrillation, and nodal tachycardia.

## 4. Conclusions

The total venom of the snake *L. acrochorda* caused the following cardiotoxic manifestations: Increased P and T wave amplitude and increased QT interval duration, due to possible electrolyte imbalance such as hyperkalemia, ischemia, or acute myocardial infarction.

*L. acrochorda* venom caused an increase in the levels of the CK enzyme (rhabdomyolysis) and its CK-MB fraction, due to cardiac myocyte damage and cardiac muscle necrosis, suggesting myocardial infarction.

In addition, it caused cardiac tissue damage such as interfibrillary and perivascular edema, cardiocytolysis, and contraction bands.

After the injection of SVLa into Rats, the alterations in cardiac tissue increased over time.

## 5. Materials and Methods

### 5.1. Obtaining the Venom

The venom was extracted manually from four adult snakes, making no distinction with respect to sex, of the species *L. acrochorda* and kept at the Biomedical Research Center of the University of Cauca (CIBUC). Once the venom was extracted, it was frozen with liquid nitrogen, lyophilized, and stored at −20 °C until the time of use.

### 5.2. Experimental Animals

For this study, juvenile male Wistar rats (*R. norvegicus*) weighing 240 g (±20 g) were used, which were also supplied by CIBUC.

The animals were kept in polypropylene boxes, acclimated to a constant temperature (22.0 ± 0.5 °C), brightness (12 h light/dark cycle), humidity, and controlled air circulation, receiving standard food and water *ad libitum*. All procedures were carried out in accordance with the standards of the National Council for the Control of Experiments with Animals (CONCEA) and were submitted for the approval of the Ethics Committee for Scientific Research of the University of Cauca. For all experiments, different doses of SVLa (3.22 μg/g, 6.43 μg/g, 12.86 μg/g) of *L. acrochorda* venom, with a value of 16.08 µg/g, were applied intraperitoneally to the biomodels.

### 5.3. Electrocardiographic Recordings

Electrocardiographic recordings were taken with puncture electrodes for 5 min from 20 biomodels that were anesthetized with sodium pentobarbital (60 mg/kg) [14]. In the experiment, 5 blocks were made, and, for the control group, the same recordings were used, but the biomodels were injected with 0.9% saline as a control. The recordings were obtained on an 8-channel ADinstruments PowerLab polygraph device, using the DII frontal plane bipolar derivation.

### 5.4. Determination of Levels of Cardiac Enzymes CK and CK-MB

After recording the corresponding data following the inoculation of the subdoses of venom, surgeries were performed on the rats, during which blood samples were taken from the descending vena cava using a 20 G intravenous catheter. The blood samples were collected in serum separator tubes (IMPROMINI microtainer, yellow cap gel and clot activator 0.5 mL), in order to facilitate the separation between serum and cellular content. Subsequently, the blood samples were centrifuged for 15 min at 3500 rpm. The serum samples were obtained using a suction method and were analyzed in the BTS-310 equipment from Byosistems was used.

### 5.5. Histological Analysis

After obtaining the blood samples, the cardiac tissue was extracted from each of the biomodels. Transverse sections of the cardiac muscle were prepared for fixation for 48 h in 10% buffered formalin, followed by the dehydration phase in ascending alcohols. Clarification or diaphanization of the samples was carried out. They were then infiltrated in paraffin at 59 °C. Finally, embedding was performed in a paraffin block, the sections (4 µm) were made using a microtome, and each slide was stained with hematoxylin-eosin. Finally, they were analyzed under an optical microscope to determine the alterations caused by the venom in the cardiac tissue.

### 5.6. Statistical Analysis

A completely randomized experimental design and a design with randomized blocks were employed, for which 20 male Wistar rats (*R. norvegicus*) were used, homogeneous in strain, age, weight, and sex. These were randomly distributed into four groups: three experimental groups (3.22 μg/g, 6.43 μg/g, 12.86 μg/g of SVLa) and one control. For histological comparisons, the Chi-squared test was applied. For the statistical analysis, the BioEstat version 5.3 GraphPad Prism version 9 programs were used. The analyses were conducted by hypothesis contrast, with a prior study of adjustment to the normal curve. The graphs were created using the GraphPad Prism version 9 program.

## Data Availability

All data are reported in the manuscript.
